# Recurrent Fungemia Due to Candida auris

**DOI:** 10.7759/cureus.62478

**Published:** 2024-06-16

**Authors:** Emma M Griffith, Christopher Marsalisi, Jorge Verdecia, Stefanie R Buchanan, Michael A Goulart

**Affiliations:** 1 Internal Medicine, University of Florida College of Medicine-Jacksonville, Jacksonville, USA; 2 Infectious Diseases, University of Florida College of Medicine-Jacksonville, Jacksonville, USA; 3 Infection Prevention, University of Florida Health, Jacksonville, USA

**Keywords:** candidemia, infection prevention and control, immunocompromised patients, anti-microbial resistance, fungemia, candida auris

## Abstract

We present a case of recurrent multidrug-resistant *Candida* *auris* (*C*. *auris*) in a patient who required multiple hospitalizations. The patient’s case was complicated by interval admissions to the intensive care unit for septic and hypovolemic shock for 12 months to manage *C*. *auris* fungemia. Despite adequate isolation precautions and appropriate antifungal treatment, this case demonstrates the profound implications of this emerging pathogen, specifically regarding invasive infections. Moreover, *C*. *auris* is rapidly becoming known as a multidrug-resistant organism, which limits treatment options and thus contributes to high mortality.

## Introduction

Discovered in 2009, *Candida auris* is a clinically significant yeast due to high mortality rates of those infected and increasing resistance to antifungal drugs [1]. More recently, media coverage and literature have illustrated the hazards of such an organism, especially in immunocompromised populations. Colonization can occur after hospitalization, but invasive infections with *C. auris* occur more frequently in patients who require intensive care. As with other species of *Candida*, known risk factors include immunocompromised status, indwelling catheters or devices, renal disease, older age, and use of broad-spectrum antibiotics [2]. This case was previously presented as a meeting abstract at Florida Chapter American College of Physicians (ACP) Fall 2023.

## Case presentation

A 48-year-old female with a past medical history of traumatic brain injury complicated by incomplete spastic quadriplegia, seizure disorder, Roux-en-Y gastric bypass, and jejunal-enterocutaneous fistula at prior gastrostomy-jejunostomy tube site was admitted for management of hypotension due to high output fistula as well as electrolyte abnormalities. No history of tobacco, illicit drug, or alcohol use was reported. Of note, she had fungemia due to *C*. *auris *10 months previously, which was attributed to the presence of a central venous catheter and successfully treated with catheter removal and 14 days of caspofungin. Multiple repeat blood cultures were negative for growth after completing the treatment course.

During this admission, she was unable to tolerate oral intake of food due to a high-output jejunal fistula, so she was started on total parenteral nutrition (TPN) via a peripherally inserted central catheter (PICC). Enhanced contact precautions were maintained, and she remained in a private room. She developed acute encephalopathy and a fever of 102.6˚F, but her PICC had no signs of infection upon examination. Infectious workup was notable for positive aerobic blood cultures with *Candida*, specifically two sets of cultures with one set being from the PICC in addition to catheter tip culture. The yeast was further speciated via matrix-assisted laser desorption/ionization-time-of-flight (MALDI-TOF) mass spectrometry to *Candida auris*. Due to her fungemia, her PICC was removed, and she was started on caspofungin. Susceptibility testing via broth microdilution assay revealed resistance to fluconazole and flucytosine as shown in Table 1. She then developed hypotension refractory to fluid resuscitation. She was transferred to the intensive care unit for management of mixed hypovolemic and septic shock in the setting of gastrointestinal losses and *C. auris* fungemia. Repeat aerobic blood cultures grew extended-spectrum beta-lactamase-producing and carbapenem-resistant *Klebsiella pneumoniae*, which was treated with 14 days of ceftazidime-avibactam. Transthoracic echocardiography showed preserved ejection fraction, but no signs of endocarditis such as vegetations. Repeat blood cultures were negative for growth. She was stabilized off vasopressors and then transferred back to progressive care with the resumption of TPN via PICC.

**Table 1:** Susceptibility of C. auris strain from the patient's blood cultures

Drug	Minimum inhibitory concentration (μg/ml)
Amphotericin B	1
Caspofungin	0.12
Fluconazole	>=256
Flucytosine	>=64

**Figure 1:**
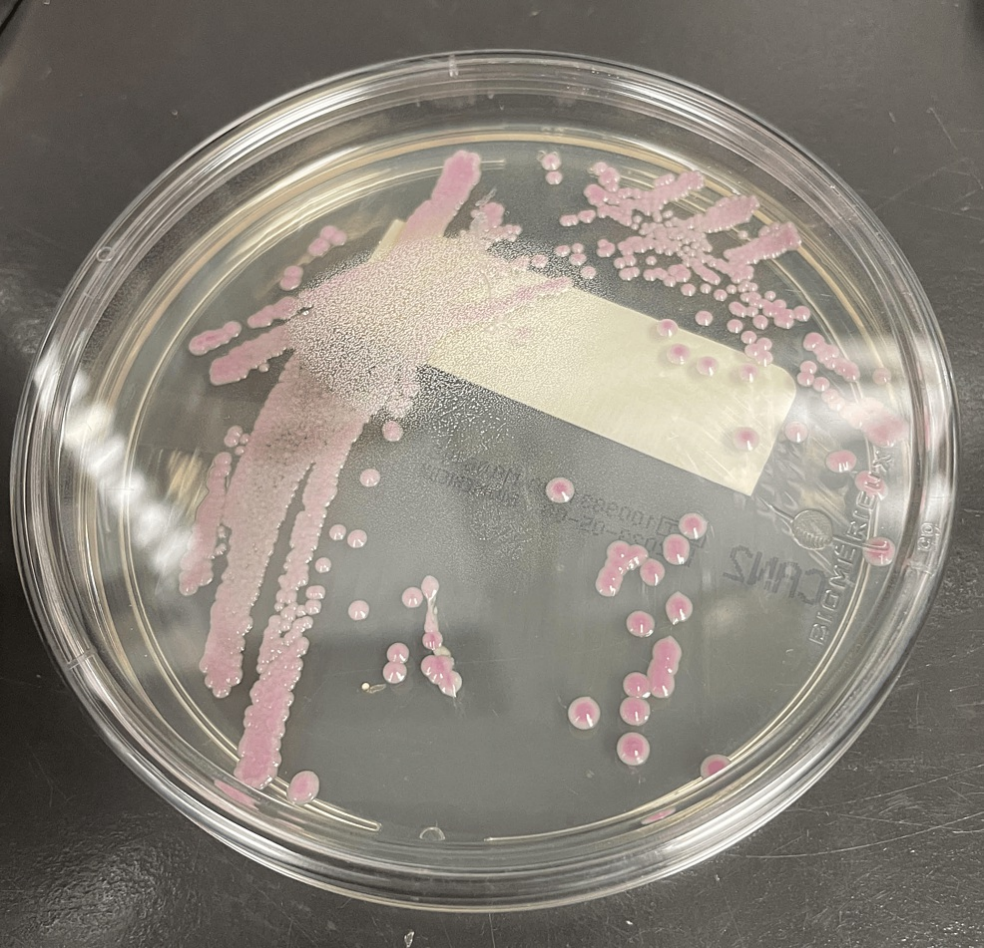
Blood culture exhibiting growth of Candida auris

Two weeks later, she had another febrile episode, and repeat blood cultures grew *C. auris, *which is* *shown in Figure 1*. *Her PICC was removed. She was started on micafungin based on further susceptibility testing. 

After family discussion, she was discharged to inpatient hospice due to multiorgan failure including shock liver and anuric renal failure prior to completion of her treatment with micafungin. Her renal failure was partially attributed to intravenous contrast exposure for imaging studies during her admission. 

## Discussion

This case demonstrates reinfection with *C. auris* despite appropriate therapy and enhanced contact precautions. Since 2013, over 5,000 cases have been reported to the Centers for Disease Control and Prevention (CDC). It has been increasing in incidence and is associated with significant morbidity and mortality leading to the “urgent antimicrobial resistance threat” label by the CDC due to resistance to common antifungal medications [3-4]. Controlling the spread of* C. auris* in healthcare facilities is challenging despite compliance with infection control measures due to limited effective disinfectants as well as its formation of biofilm leading to an ability to survive on surfaces for months [5]. Per the CDC, isolation precautions in healthcare settings should include staff use of gowns and gloves, handwashing with soap and water, and private rooms. Isolation should be maintained in patients with any history of *C. auris*, as colonization is frequent and there is no effective decolonization protocol or expiration date for isolation [4]. Diagnosis of *C. auris* is typically via culture with subsequent testing such as sequencing or MALDI-TOF mass spectrometry. In this case, aerobic blood cultures grew *Candida*, which was then speciated via MALDI-TOF mass spectrometry. Treatment is reserved for invasive infections such as fungemia, not for asymptomatic epidermal or urinary colonization. This case was unique due to its resistance to amphotericin B but similar to other reported cases in its susceptibility to echinocandins and resistance to azoles [6-7]. Resistance patterns vary based on geographic region as well as prior exposure to antimicrobial medications. Further investigation regarding isolation precautions is needed, and central catheter use should be limited in patients with known history of *C. auris*.

## Conclusions

Reinfection with* C. auris* can occur in colonized patients who underwent invasive procedures such as central line placement or developed immunocompromised status despite adherence to infection prevention guidelines per the CDC. Current recommendations include lifelong isolation precautions in these patients due to lack of decolonization protocol. There are limited efficacious antifungal medications for the treatment of this microorganism due to its increasing antimicrobial resistance. This case enhances the available body of literature and highlights the need for further investigation into infection prevention of *C. auris*.
